# Evaluation of an activity monitor for use in pregnancy to help reduce excessive gestational weight gain

**DOI:** 10.1186/s12884-018-1941-8

**Published:** 2018-07-31

**Authors:** Paul M. C. Lemmens, Francesco Sartor, Lieke G. E. Cox, Sebastiaan V. den Boer, Joyce H. D. M. Westerink

**Affiliations:** 1 Philips Research, High Tech Campus 34, 5656 AE Eindhoven, the Netherlands; 2 Eindhoven University of Technology, Het Eeuwsel, 5612 AZ Eindhoven, the Netherlands

**Keywords:** Activity monitor, Heart rate, Accelerometer, Validation, Energy expenditure, MET

## Abstract

**Background:**

Excessive weight gain during pregnancy increases the risk for negative effects on mother and child during pregnancy, delivery, and also postnatally. Excessive weight gain can be partially compensated by being sufficiently physically active, which can be measured using activity trackers. Modern activity trackers often use accelerometer data as well as heart rate data to estimate energy expenditure. Because pregnancy affects the metabolism and cardiac output, it is not evident that activity trackers that are calibrated to the general population can be reliably used during pregnancy. We evaluated whether an activity monitor designed for the general population is sufficiently accurate for estimating energy expenditure in pregnant women.

**Methods:**

Forty pregnant women (age: 30.8 ± 4.7 years, BMI: 25.0 ± 4.0) from all three trimesters performed a 1-h protocol including paced and self-paced exercise activities as well as household activities. We tracked reference energy expenditure using indirect calorimetry and used equivalence testing to determine whether the estimated energy expenditure from the activity monitor was within the limits of equivalence.

**Results:**

Overall we found an averaged underestimation of 10 kcal (estimated energy expenditure was 97% of the reference measurement). The 90% CI for the cumulative total energy expenditure was 94–100%. The activities of self-paced cycling, household activities, stair-walking, and yoga had one of their equivalence boundaries outside a 80–125% range of equivalence; for exercise on a cross-trainer, for self-paced and fixed-pace walking, fixed-paced cycling, and resting, the estimations were within the limits of equivalence.

**Conclusions:**

We conclude that the activity monitor is sufficiently accurate for every-day use during pregnancy. The observed deviations can be accounted for and are acceptable from a statistical and an applied perspective because the positive and negative deviations that we observed cancel out to an accurate average energy expenditure over a day, and estimations during exercise are sufficiently accurate to enable coaching on physical activity. The positive and negative deviations themselves were relatively small. Therefore, the activity monitor can be used to help in preventing excessive weight gain during pregnancy by accurately tracking physical activity.

## Background

Excessive Gestational Weight Gain (eGWG) is defined as a weight gain of more than 10 kg for women of normal pre-pregnancy weight, more than 9 kg in pregestationally overweight women, or a more than 6 kg weight increase in pregestationally obese women [1, 2]. eGWG is an increasingly prevalent health risk for pregnant women that affects more than 50% of all pregnancies in the United States (US) [3, 4]. It has been shown to be an independent risk factor for multiple medical conditions including gestational diabetes mellitus (GDM), gestational hypertension and pre-eclampsia [5, 6]. Physical *inactivity* is the main contributors to eGWG, and is relatively common in our increasingly obesogenic environment [7, 8]. In contrast, physical *activity* has been shown to have protective effects against GDM and pre-eclampsia [9–11], while not affecting fetal growth [11] or triggering premature delivery [11].

Concerning physical activity during pregnancy, guidelines recommend a minimum of three 15-min sessions per week up to a maximum of four 30-min sessions per week at a moderate to hard exertion level [2, 12, 13]. These exertion levels are considered safe, provided that there is no medical contraindication [2]. The targets that the various guidelines propose vary from 16 to 28 Metabolic Equivalent of Task (MET) hours per week (1 MET is equivalent to energy expenditure during rest). For a 71 kg person on a schedule of 4 days of exercise per week, this corresponds to an energy expenditure between 300 to 525 kcal during exercise [12]. However, these recommendations are often not met [14, 15] partly because these abstract guidelines in terms of METS or kcals are often difficult to translate into real-life exercise targets that, cumulatively over the duration of the pregnancy, will result in achieving sufficient physical activity to prevent eGWG.

A possible solution would be to accurately and objectively measure expended energy during physical activities, and to provide feedback about it in terms of activity minutes of activity or energy expended [16–18] in free-living settings [19, 20]. Accelerometer and heart-rate based activity monitors have become available that provide such an accurate yet unobtrusive estimation of energy expenditure [21–25]. The combination of accelerometer and heart-rate provides improved accuracy compared to questionnaires [20] as well as compared to accelerometer-only or HR-only estimations of energy expenditure [25, 26]. Such products could help pregnant women to determine whether their expended energy matches the proposed physical activity guidelines and become more compliant to them. It is paramount that the estimated energy expenditure is sufficiently accurate to enable appropriate guidance to minimize eGWG when physical activity is observed to be insufficient.

To our knowledge, however, none of the consumer-grade devices exploiting a combination of accelerometer and heart rate based estimation of energy expenditure has been validated for use during pregnancy. This is remarkable because it is known that a pregnancy has distinct effects on physiological and metabolic processes [27, 28]. For instance, resting metabolic rate (RMR) has been shown to be significantly different in pregnant women compared to matched controls [29], and even within pregnancy significant differences between the first, second and third trimesters have been observed on, for instance, cardiac output [30, 31]. Thus, a validation on the general population might not suffice.

Here, we aim to validate whether an existing activity monitor combining accelerometry and heart rate information to estimate energy expenditure, is sufficiently accurate to be used in guiding and monitoring pregnant women in achieving the activity levels set out in the guidelines to prevent or minimize the effects of eGWG and its comorbidities. Because of the noted differences on physiological parameters over trimesters, the validation was performed on women of all gestational ages.

## Methods

### Participants

**Fig. 1:**
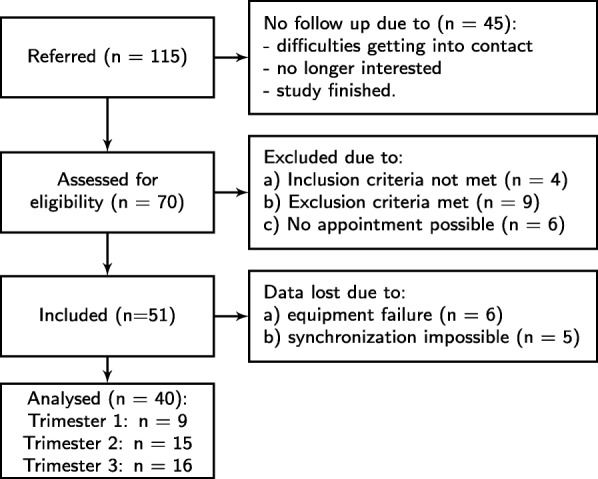
Participant flow in the study

We included 51 pregnant women from all gestational ages (12–35 weeks pregnant). Participants were required to be in primary care which excluded high-risk pregnancies. Of this set of 51 participants, the data of 40 participants were used in the statistical analyses (see Fig. 1). The data of eleven participants could not be used due to equipment failure or inability to properly synchronize the data from the sensors. The mean age of the 40 participants was 30.8 ± 4.7 years, average height was 169.5 ± 7.8 cm, and the average weight was 71.7 ± 12.6 kg. For each participant we estimated VO_2max_ using the equation in Fig. 1 of Sady et al. [32] using HR_60W_ and VO_2,60W_ from the last minute of the 6-min cycling activity in the protocol (see below). The average value for VO_2max_ was 33.0 ± 6.8 ml/kg/min. The study was approved by our institutional review board (Philips Research IRB dossier ICBE-2-3895; formal medical-ethical review was waived) and written informed consent was obtained from all participants prior to collecting any data. Participants were recruited and referred by a local recruiting agency.

### Materials

We used two devices to measure and estimate energy expenditure. The reference measurements were taken by a Cosmed K4b^2^ calorimeter (Cosmed, Italy) that uses a breath-by-breath pulmonary gas exchange (VO_2_, VCO_2_) analyzer to calculate (total) energy expenditure [33]. The K4b^2^ was calibrated according to the manufacturer’s instructions by first performing a room-air calibration, then a reference-gas calibration using a gas tank with a 16% O_2_ and 5% CO_2_ mix, and finally a flow-turbine calibration using a 3.0 L syringe.

The second device was a watch (referred to as Optical Heart Rate Monitor, OHRM) that was worn on the participants’ left wrist. The OHRM is based on the Philips Cardio and Motion Monitoring Module (CM3-Generation-1) that is an accelerometer and optical heart rate sensor module developed by Philips [21, 34]. It estimates energy expenditure using contributions of measured heart rate and accelerometry. Additional devices were employed to make sure that protocol timing and synchronization of the various data streams was possible. One of these devices was a Garmin Forerunner 620 that we used as master clock and to set markers at the beginning and end of each activity.

### Protocol

Participants were asked to fill out the International Physical Activity Questionnaire (IPAQ) to evaluate their baseline physical activity level of the last seven days. Age, height, weight and gestational age were recorded. Then a series of physical activities was performed that each lasted for 1–3 min and one activity lasting for 6 min (see Table 1). Each activity was followed by 1–3 min of (seated) rest to prevent fatigue. The durations of activities and rest were based on striking a balance between the need for a minimum duration to achieve steady state (based on pilots and earlier studies) and keeping the overall duration and exertion level acceptable for pregnant women. During the indoor laboratory protocol, exercises with high and low intensity were alternated to balance overall physical exertion. Based on pilots and earlier studies, we defined high intensity activities as those where participants were expected to achieve a heart rate of at least 60% of maximal heart rate which Zavorsky and Long set as definition of vigorous activity during pregnancy [2]. Half of the participants performed the indoors activities in the reversed order. During the outdoor part, cycling and various forms of walking were required. The total duration of the activities was 61 min, excluding rest and a break between the indoor and outdoor part. Heart rate was monitored continuously to ensure participants did not exceed 85% of their maximal HR.

**Table 1:** Overview of the protocol that was used in the study with durations of the activity and rest immediately after the activity indicated in square brackets

Heart rate at rest [5]	
Indoor activities	
1. Stacking groceries [3, 1]	
2. Desk work [2, 1]	
3. Vacuuming [3, 2]	
4. Sitting resting [3, 1]	
5. ^a^Cycling fitness test (60 W) [6, 5]	
6. ^a^Walking treadmill - 3 km/h – 0% incline [3, 2]	
7. Standing resting [1, 1]	
8. ^a^Walking treadmill - 5 km/h - 0% incline [3, 2]	
9. Folding towels [3, 1]	
10.^a^Walking treadmill - 5 km/h - 0% incline - carrying 4 kg [3, 3]	
11. Cooking or Washing dishes [3, 1]	
12. ^a^Walking treadmill - 3 km/h - 5% [3, 3]	
13. Cleaning table [3, 1]	
14. ^a^Cross trainer - 60 W [3, 5]	
15. Yoga [3, 1]	
Switch to self-paced/outdoor activities	
16. Walking upstairs (indoors) [1, 2]	
17. Walking downstairs (indoors) [1, 2]	
18. Walking, hands free [2, 1]	
19. Walking, hands in pockets [2, 1]	
20. Walking, carrying a bag [2, 1]	
21. Cycling [3, 2]	

After step 15, a pause of 5–10 min was required to replace the battery of the K4b^2^ and to prepare participants for doing the self-paced activities that were executed indoors and outdoors. Before the activities, all participants started with a 5-min measurement of heart rate at rest so all participants started at a similar steady state in rest. Note that, due to weather conditions, for some participants, the self-paced activities were performed indoors. Also note that cooking and dishwashing (11) were “role-playing” activities mimicking the actual activities. Activities marked with an 'a'  were fixed paced activities enforced by either setting a specific speed of the respective exercise machine or by monitoring by the study assistant

### Data synchronization, preprocessing, and statistical analysis

We used a series of steps to synchronize all separate data sets. Because most devices did not have the option to place a marker in their data to synchronize the devices, we used a 1-min shaking protocol during which all sensors containing accelerometers were shaken vigorously to introduce a signal in their accelerometer data that could be easily recognized. This shaking protocol was performed before the sensors were placed on the participants.

We started the preprocessing with converting the K4b^2^ data by linear interpolation from a breath-by-breath frequency to a time-series with a sampling frequency of 1 Hz. Then we synchronized the data sets first based on heart rate and subsequently based on accelerometer data using cross-correlation techniques with possible fine-tuning based on visual assessment. We used the Garmin as master clock and synchronized the data of the ORHM and the K4b^2^ to the Garmin’s heart rate.

Next, we extracted the cumulative total energy expenditure (TEE in kcal) for both devices from the recorded and estimated energy expenditure at the end of the laboratory protocol. We calculated the average (total) energy expenditure (kcal/h). The average bias (kcal/h) was determined by subtracting the reference from the estimated energy expenditure. Mean absolute percentage error (MAPE) for each individual activity was calculated by extracting the data for the individual activities from the synchronized data using the markers and their associated time stamps that we set using the Garmin. Finally, we calculated for each activity, 10-s non-overlapping windows of averaged TEE to compute root-mean-squared errors (RMSE) on the differences between reference and estimated expenditure. We chose non-overlapping windows because this was consistent with how one would use the averaged TEE samples to calculate the cumulative TEE over each activity or even the full protocol.

We based the statistical analysis on equivalence testing [35] and tested whether the ratio of the OHRM-based estimated energy expenditure and the K4b^2^-based reference measurements thereof was within an acceptable interval around 100% [36, 37]. As acceptable interval, we used the 20% margin that has international consensus [38–40] but also used a tighter 10% margin. We determined equivalence based on the differences in log-transformed energy expenditure because this is mathematically equivalent to a ratio of untransformed values. This transformation is required because the equivalence testing procedure only works on differences. Therefore, a symmetric 20% margin translates as a range of values from 80 to 125% (= 1/0.8) for the assessment of equivalence in log-transformed units [41]. That is, when the 90% confidence interval for the ratio of the OHRM’s estimations and the measured TEE of the K4b^2^ was within the interval ranging from 80 to 125%, we declared the monitor’s estimation as equivalent.

We implemented the equivalence testing using the two one-sided tests (TOST) procedure. It requires calculating (100 – 2α) = 90% confidence intervals (90% CI) [42, 43] for the observed difference between reference and estimated scores to determine whether that 90% CI falls within the pre-established equivalence margin. Tryon and colleagues [44, 45] have analytically shown that the TOST procedure and the visualizations that we use are equivalent. Therefore, we refrained from using *p*-values throughout the equivalence analysis, but instead we focused on visualizations of the observed confidence intervals against the regions of equivalence of 80–125% as well as a more restrictive 90–111% interval, concluding equivalence only if the observed confidence interval was fully included in the 80–125% region of equivalence. Note that we carried out the analysis on differences of log-transformed energy expenditure but that the visualizations are presented on back-transformed ratios expressed as percentage of the reference.

In addition to equivalence tests, we also calculated bias (difference in kcal/h) between the OHRM and indirect calorimetry from the K4b^2^, and errors (in terms of mean percentage errors, and mean absolute percentage errors) for total energy expenditure. We used the visual technique of Bland-Altman plots [46, 47] to determine the degree of agreement, and to evaluate the bias for aspects like proportional error (heteroscedasticity). In addition, Bland-Altman plots provide an overall view of the data, including outliers, and enables, via the limits of agreement (LOAs), the assessment of the relevance of potential outliers by showing whether the LOAs are influenced mostly by the general quality of the agreement or also by outliers. Thus a Bland-Altman plot provides more information than, for instance, a concordance correlation coefficient.

Based on similarity of activities and whether activities were self-paced or not, we created nine clusters of activities: cycling (paced, indoors), cycling (self-paced, outdoors), walking (paced, indoors), walking (self-paced, outdoors), resting, stair walking, household activities, yoga, and cross training; The latter two clusters actually comprised a single activity. We based our statistical analyses on these activity clusters.

## Results

**Fig. 2:**
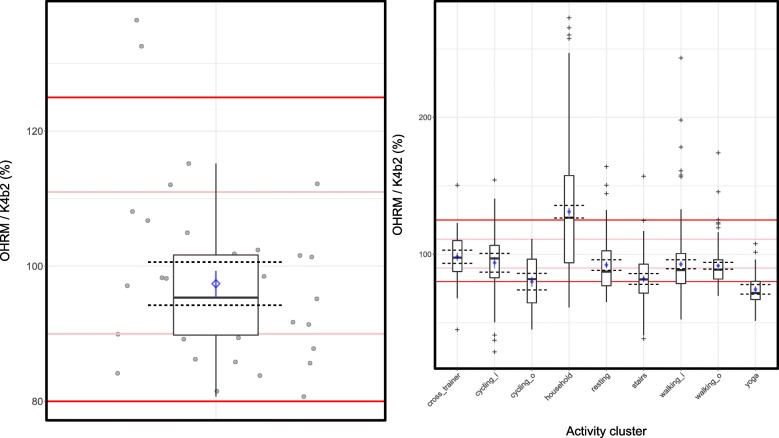
Average estimated TEE (kcal/h) as percentage of the reference; overall and per activity cluster. Boxplot of the cumulative TEE of the entire lab protocol with jittered raw data points from participants’ individual cumulative TEE as percentage of reference TEE (left-hand panel; with mean and standard error in blue) and (right-hand panel) per activity cluster (without raw data points and plusses for outliers; mean and standard error in blue). Dashed horizontal black lines indicate the 90% confidence interval around the averaged cumulative TEE ratio. Solid pink horizontal lines reflect the 90–111% limits and the red lines reflect the conventional 80–125% limits of equivalence. In the right-hand panel, in the x-axis labeling, a suffix “i” concerns indoor activities at a fixed pace on the treadmill or ergometer; the “o” suffix concerns data from outdoor activities that were self-paced

We found that the estimations of TEE by the OHRM averaged over all activities were at 96.8% (SD: ± 12.0) of the reference measurements (90% CI: 93.8–99.8%). It was clear that the 90% confidence interval around the OHRM-estimated cumulative TEE for the duration of the lab protocol fell well within the boundaries for equivalence of the conventional 80–125% case as well as of the more restrictive 90–111% (see the left-hand panel in Fig. 2).

Next, we determined whether the average TEE estimation from the OHRM was equivalent to the averaged reference measurements from the K4b^2^ for each cluster of activities (see Fig. 2, right-hand panel). This shows that the 90% CI’s for the walking activities (fixed pace as well as self-paced), resting, fixed pace cycling, and the cross training were well within the conventional 80–125% boundaries of equivalence, although the OHRM’s estimations were too high or too low for some participants. Only the cross-training activity achieved an accuracy with a 90% CI that was within the more restrictive 90–111% boundary.

We found that for four activity clusters, the upper or lower limit of the 90% CI of the OHRM’s estimations were higher or lower than the 80–125% range of equivalence. For the household activities we observed that the upper CI limit was larger than 125% and that, with the median approximately equal to the 125% limit, a little over half of the data fell above the 125% equivalence limit. This indicated that the OHRM overestimated energy expenditure for this activity cluster. On the other hand, we observed that for outdoor (self-paced) cycling as well as stair walking the lower limit of the 90% CI was below the 80% equivalence limit, reflecting estimations that were too low compared to the K4b^2^’s reference measurement. The yoga activity was the only activity cluster that was completely outside of the 80–125% equivalence boundaries. There, the estimations were too low compared to the reference measurements.

**Fig. 3:**
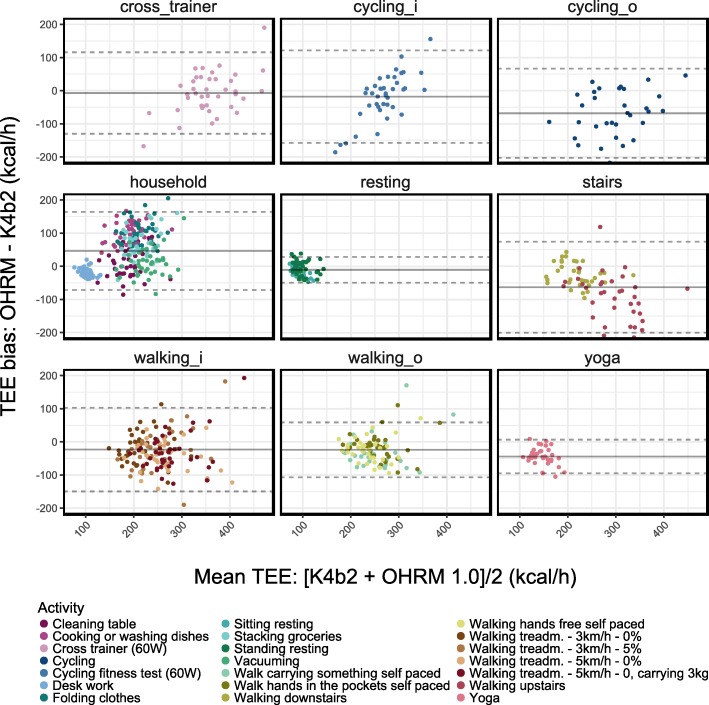
Bland-Altman plots of TEE (kcal/h) for each activity cluster. Solid grey lines indicate the cluster’s average TEE bias and dashed lines reflect the 95% limits of agreement (LoA). Colored dots are participants’ averaged TEE biases for each activity. Average biases and LoA’s were calculated for the collective data of each cluster. In the panel labels, a suffix “i” concerns indoor activities at a fixed pace on the treadmill or ergometer; the “o” suffix concerns data from outdoor activities that were self-paced

To study whether the observed deviations in estimated energy expenditure could be detrimental to, for instance, physical activity programs to reduce (e)GWG, we considered the deviations in terms of kcal/h because those programs aim for a specific exercise intensity to expend a certain amount of kilocalories. A series of Bland-Altman plots for the activity clusters showed that the bias (the difference) between TEE estimation and the TEE reference measurement ranged, on average, from − 68.0 kcal/h (SD: 68.7; self-paced cycling) to + 46.2 kcal/h (SD: 60.1; household activities; see Fig. 3). The best estimations of TEE of the OHRM were obtained during the cross-trainer activity and during rest with average deviations as small as − 7.1 (62.7) kcal/h and − 10.3 (19.8) kcal/h, respectively.

The Bland-Altman plots showed several interesting patterns: for instance, the marked difference in estimation accuracy for upstairs versus downstairs walking. Whereas the estimations for the latter were quite accurate with an error close to zero on average, the estimations for the former were too low compared to the reference measurements and this underestimation resulted in the overall underestimation for the activity cluster of stair walking that we observed in Fig. 3 (right-hand panel).

Another striking pattern in the Bland-Altman plots was the concentrated cluster of data points in the lower left part of the (middle-left) panel of Fig. 3 that concerned the household activities comprising (computer) desk work, stacking groceries, vacuuming, folding clothes, cleaning a table, and cooking and dish washing. This cluster of data points was from the desk-work activity that, relative to activities like grocery stacking, cooking, and dish washing, was a static activity with minimal hand and body movements resulting in very low energy expenditure. The other household activities were characterized by many repetitive hand movements and little body movement resulting in an overestimation by the OHRM.

The third interesting pattern is the one related to the indoor cycling (top-middle panel) that seems to show a correlation between the bias and TEE, indicative of a proportional error [46, 47]. However, this seems to be caused largely by the four data points around the LoA that overemphasize the proportional error. When we removed these data points, the apparent proportional error was no longer evident. Overall, we did not find further consistent or considerable flaws in the TEE-estimations by the OHRM indicated by the absence of patterns in the Bland-Altman plots.

**Fig. 4:**
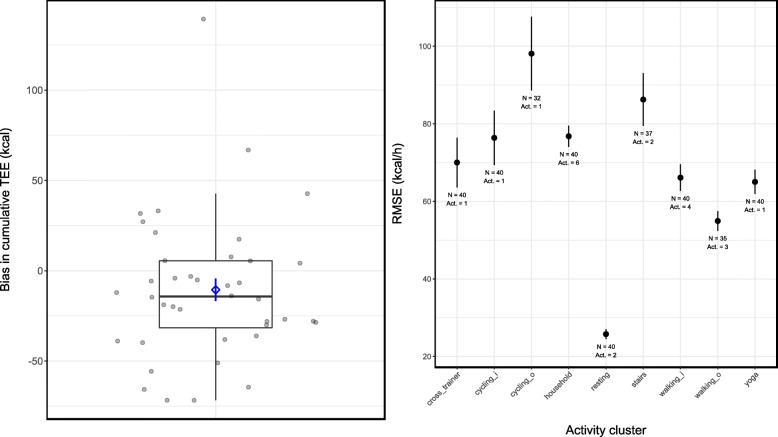
Bias in cumulative TEE, and RMSE of cumulative TEE (kcal) separately for each activity cluster. Box and whiskers plot, with participants’ individual data jittered, of the overall difference between the OHRM’s TEE estimations and the K4b^2^ reference measurement with the averaged bias with standard error in blue (left-hand panel), and RMSE’s for each activity cluster (with standard errors in error bars; right-hand panel). In the x-axis labels of the right-hand panel, a suffix “i” concerns indoor activities at a fixed pace on the treadmill or ergometer; the “o” suffix concerns data from outdoor activities that were self-paced. Note that the panels have different y-axis ranges. Also note that in the right-hand panel the sample size is different between activity clusters as indicated per cluster with the sample size (N) and the number of activities within each cluster (act)

From the biases calculated for the Bland-Altman plots, we determined that the average overall bias was − 10.6 kcal (Fig. 4, left-hand panel; without the outlier − 14.4 kcal) for the 61 min protocol which amounted to a mean percentage error of − 2.6% and a mean absolute percentage error of 9.4%. This indicated that on average the OHRM’s estimations were 10.6 kcal too low which was a − 3% error. RMSE’s for each activity cluster ranged from 26.0 kcal/h (0.35 MET; resting activities) to 98.1 kcal/h (1.3 MET) for the self-paced cycling activity.

## Discussion

Commercial solutions for estimating energy expenditure exist but to the extent of our knowledge have not been validated for use by pregnant women. We performed a study to determine whether the OHRM activity monitor [21], that estimates energy expenditure based on accelerometer and heart rate data at the wrist, is sufficiently accurate for use during pregnancy. This monitor has been developed based on data from the general population and it is known that a pregnancy changes some of the key biophysiological parameters upon which the OHRM builds its estimations [27–30]. In a protocol that combined a series of paced and self-paced activities mimicking exercise activities as well as everyday life activities, participants used the OHRM and the activity monitor’s estimated energy expenditure was compared against reference measurements from indirect calorimetry.

The data that we have gathered show that for cumulative total energy expenditure (TEE) the OHRM’s estimations are on par with the reference K4b^2^’s measurements for a range of equivalence of 90–111%, which is stricter than the conventional 80–125% range [38–40]. On average, the estimated cumulative TEE is at 97% of the reference value from the K4b^2^. When considering clusters of highly similar activities, on average, the estimations are within the conventional limits of equivalence of 80–125% for cross-trainer activities, indoor cycling, resting, and indoor and outdoor walking.

When converting the observed errors into METS, we find values that are below or a little over 1 MET which is equivalent to the energy expenditure at rest. For a 73 kg person, the largest errors in TEE estimation equated to − 0.91 MET (self-paced cycling) and 0.62 MET (household activities) and around 0.1 MET for the smallest errors of cross training and resting. These small errors should enable appropriate guidance on physical activity in a program to minimize or prevent excessive weight gain during pregnancy.

However, four activities had 90% CI’s for their averaged TEE that are partially or fully outside the region of equivalence. For outdoor cycling, stair walking, and yoga we observed underestimations whereas energy expenditure during household activities was overestimated. A possible reason for the overestimation above the 125% border of equivalence (see Fig. 2) of TEE for the household activities is that the activities that participants performed involved relatively stationary yet manually intensive activities. Because the OHRM was positioned at the wrist, our hypothesis is that the overestimation by the OHRM occurs because the contribution of the accelerometer data in the energy expenditure estimation is too high due to the mostly manual activities with intensive hand and wrist movements whereas the reference measurement hardly changes due to the low overall intensity of the activities. This pattern of overestimation due to manually-intensive activities is visible in the Bland-Altman plot (see Fig. 3) for the household activities, with a large cluster of data points with a positive bias of about 50 kcal/h and a spread that ranges from − 72 to 163 kcal/h.

The estimations for self-paced cycling were lower than the 80% border of equivalence. The speed data from the GPS sensor that the participants wore during the outdoor activities showed an average speed of about 12.6 km/h during cycling. This is a low speed for cycling that may have further reduced the already limited movement of the wrist and that may have resulted in an accelerometer contribution that is too low, thus resulting in underestimation of energy expenditure. An additional explanation is proposed by Hendrikx and colleagues [34] that also applies to our study due to (also) having been performed during winter time. Their explanation revolves around cold outside temperatures that result in (additional) vasoconstriction that, in turn, excessively reduces the amount of time in which the estimated heart rate is within a reasonable distance from the reference heart rate thus increasing the difference between reference and estimated energy expenditure.

The last activity for which the 90% confidence interval was partially outside of the 80–125% range of equivalence was stair walking. The Bland-Altman plot for the stair walking activities in Fig. 3 shows a clear difference in estimation accuracy between walking upstairs (when the OHRM underestimated energy expenditure) versus walking downstairs (when the OHRM was on par with the reference energy expenditure). We speculate that this difference is related to not having properly controlled whether participants used the hand rail of the stairs. That is, when the wrist is hardly moving due to using the hand rail the accelerometer based contribution to energy expenditure estimation is too low.

The one activity having a 90% confidence interval fully outside of the equivalence region was the yoga activity for which the OHRM estimates TEE, on average, at 74.4% of the reference measurement. Our hypothesis for the underestimation is that the yoga activity comprised six 30-s stationary yoga positions. Due to the stationary nature of the yoga positions, the contribution of accelerometry to the estimation of energy expenditure is too low. This situation is exacerbated by the fact that holding the yoga positions is an activity of moderate to high intensity for pregnant women resulting in an increased difference between reference measurements and estimations of energy expenditure.

Overall, we find that the OHRM activity monitor, when used in pregnant women, provides energy expenditure estimations that, on average, are about 10 kcal too low for a 61 min protocol of activities, which amounts to an averaged error of − 2.6% (MAPE, 9.4%). This low overall error also highlights that although some types of activities are overestimated and others underestimated, these average out over the day to achieve an estimated energy expenditure very close to the reference value(s). Because effective prevention of eGWG is based on being sufficiently active throughout the entire day, the overall estimation error is the most relevant error to consider; additionally, the OHRM does accurately monitor energy expenditure during exercise activities. The overall underestimation is similar to the − 3% (and 10% MAPE) error reported by Hendrikx and colleagues [34] when they validated a medical class-2a activity monitor in the general population in a 48 min protocol. The high similarity of these observed errors highlights that the OHRM can be readily used in pregnant women as well as in the general population.

Our main finding therefore is that the OHRM can be reliably used in pregnant women to provide accurate lifestyle and activity coaching to prevent or minimize the detrimental effects of excessive gestational weight gain. This finding is limited by the following aspects of our study. The most important limitation is that our analyses and conclusions are based on extrapolations of data obtained during a laboratory protocol of limited duration whereas the intended use case would be a 24 × 7 scenario. Although the technology around indirect calorimetry is improving and is enabling longer recording times with smaller devices, the limitations of indirect calorimetry still preclude recording true 24 × 7 reference data. Another limitation is the fact that we did not deploy a longitudinal design involving repeated measurements of the participants in all three trimesters of their pregnancy. This would have enabled us to assess the within-person reliability of the energy expenditure estimations within and over trimesters of pregnancy. Nevertheless, our sample is representative for the Dutch population on aspects like age and BMI, and its coverage of the complete pregnancy duration.

## Conclusion

We have shown that the OHRM activity monitor accurately estimates energy expenditure for use in activity coaching for pregnant women. Overall, the data from our study show an average error of about − 3% percent. Overestimation for specific (sets of) activities cancel out with underestimations for other activities and the observed errors translate to negligible values of around 1 MET. The average error is comparable in accuracy to the average error of a similar (medical class-2a) activity monitor that is validated on the general population. We therefore conclude that the OHRM can be readily used in pregnant women to help minimize or prevent excessive gestational weight gain.

## **Abbreviations**

eGWG: Excessive gestational weight gain
GDM: Gestational diabetes mellitus
HR: Heart rate
IPAQ: International Physical Activity Questionnaire
MET(S): Metabolic equivalent of task
OHRM: Optical heart rate monitor
RMSE: Root-mean-squared error
TEE: Total energy expenditure
